# Development and validation of the adolescents’ perceptions of parental involvement scale in China

**DOI:** 10.3389/fpsyg.2025.1529173

**Published:** 2025-05-14

**Authors:** Xuerong Liu, Zheng Gong, Wei Li, Jingyu Lei, Xiaodi Han, Yaozhi Wang, Xueqian Wang, Liping Shi, Qiongzhi Zhang, Qianyu Zhang, Heng Zhang, Zhanghong Li, Jianhong Liu, Jie Gong, Zhiyi Chen, Zhengzhi Feng

**Affiliations:** ^1^Experimental Research Center of Medical and Psychological Science, School of Psychology, Third Military Medical University, Chongqing, China; ^2^Key Laboratory of Cognition and Personality, Ministry of Education, Faculty of Psychology, Southwest University, Chongqing, China; ^3^Department of Educational Sciences, Sichuan Normal University, Chengdu, China; ^4^Langfang Foreign Language School, Langfang, Hebei, China; ^5^Rongchang District Teacher Training School, Chongqing, China; ^6^Daixian Second Middle School, Xinzhou, Shanxi, China; ^7^Nanchong Psychosomatic Hospital, Nanchong, Sichuan, China

**Keywords:** adolescence, parental involvement, grounded theory, scale development, validation

## Abstract

**Introduction:**

Given the increasing academic pressures associated with intensified social competition, the role of parental involvement in education has emerged as a pivotal research area, especially within the context of Chinese culture. However, there remains a need to further delineate the characteristic dimensions of parental involvement and to develop appropriate measurement tools to quantify such involvements. By the perspective from adolescents’ reports, this study proposed a psychometric and theoretical framework for understanding Chinese adolescents’ perceptions to their parental involvement.

**Methods:**

We adopted a mixed-methods approach across three studies. In Study 1, we conducted structural interviews with adolescents aged 12 to 18, applying grounded theory to analyze their perceptions of parental involvement. Study 2 involved the creation and refinement of a measurement questionnaire through exploratory factor analysis. Study 3 validated these dimensions through confirmatory factor analysis and criterion-related validity tests on national samples.

**Results:**

Study 1 identified four preliminary characteristic dimensions of perceived parental involvement: emotional involvement, academic involvement, life involvement, and social involvement. In Study 2, we developed a four-dimensional 21-item questionnaire showing high conceptional validity. Study 3 demonstrated strong reliability and construct validity for this questionnaire across different samples.

**Discussion:**

This research provides a new understanding of the characteristic dimensions of adolescents’ perceptions of parental involvement and introduces an effective measurement tool.

## Introduction

In recent years, heightened social competition has resulted in escalating parental aspirations for their children’s achievements. Parents are increasingly resorting to diverse tactics to secure superior educational opportunities for their children, with the aim of furnishing them with a competitive advantage in the future (Benner et al., 2016; Pinquart and Ebeling, 2020). Against this circumstance, parental involvement has become a central focus for researchers. Terms like “jiwa,” which highlight parents’ substantial investment of time and resources in their children’s education and development, have entered the modern educational discourse, illustrating this growing trend. However, adolescence represents a unique phase of individual development, characterized by increased autonomy and exploratory behavior (Benner et al., 2016). While parental involvement can positively influence adolescent development, it also presents significant challenges (Day and Dotterer, 2018; Schoon and Burger, 2021). Excessive academic pressure can result in heightened psychological issues, reduced creativity, and diminished interest in autonomous learning among adolescents (Berdida, 2023; Cheng and Lin, 2023; Yao et al., 2023). Moreover, it can exacerbate educational inequality and social stratification, adversely affecting holistic development. Despite these challenges, there is a notable scarcity of research examining parental involvement from a psychological perspective, particularly in the context of Chinese culture. An in-depth exploration of the psychological connotations and characteristic dimensions of parental involvement in such cultural changes remains necessary to fully understand its impact on adolescents and guide more effective parental engagement strategies.

The concept of parental involvement was introduced in the 1960s by the U.S. government’s Head Start Program, which emphasized the equal partnership between parents and educational professionals in the education of children. This idea quickly permeated educational policies and became a fundamental component of the educational system (Wright et al., 2007). As indicated by previous studies, scholars have redefined parental involvement as a multidimensional construct encompassing parent–child interactions within the home and communication and cooperation between parents and schools (Fantuzzo et al., 2000; Jiang et al., 2023). Within the unique cultural context of China, parental involvement often reflects profound societal responsibility and high expectations, such as a strong focus on children’s education and aspirations for their future success (Liu, 2023; Xu et al., 2020). This has sparked societal debates and phenomena, with parents increasingly investing in their children’s academic pursuits, sometimes resulting in detrimental “educational competitions” (Zhang and Xu, 2023). According to social capital theory, parental involvement is seen as a process of enhancing children’s competitiveness through resource and information investment, highlighting the role of parents as primary providers of educational resources (Keith et al., 1998).

To deeply understand the impact of parental involvement, it is essential to consider not just the actual involvement behaviors but also adolescents’ perceptions of this involvement. Research indicates that adolescents’ subjective perceptions of parental involvement can significantly influence their psychological states and behavior patterns, often more so than the actual involvement itself (Korelitz and Garber, 2016). Parental involvement perception refers to how adolescents subjectively experience and interpret the level of their parents’ involvement in their lives. This concept underscores that even if parents believe they are reasonably involved in education and daily life, adolescents may perceive this involvement as insufficient or excessive. Such perceptual discrepancies can lead to tension in the parent–child relationship, potentially adversely affecting adolescents’ mental health and overall development (Liu and Zhang, 2023). Therefore, while educational research has extensively explored the positive effects of parental involvement on academic achievement, social adaptation, and psychological development (Wang and Sheikh-Khalil, 2014), there remains a pressing need to investigate its psychological connotations and characteristic dimensions from a psychological perspective. This approach is crucial for understanding the complex dynamics of parental involvement and its broader implications for adolescent development.

Parental involvement has long been recognized as a major influence on child and adolescent development, with extensive research dedicated to its assessment and effects. However, existing studies often exhibit notable limitations in the conceptualization and measurement of this construct. For example, much of the work in the field has predominantly emphasized educational or academic perspectives. The Father Involvement Scale (Finley and Schwartz, 2004) and the Mother Involvement Scale (Finley et al., 2008)were designed to retrospectively assess young adults’ perceptions of their parents’ involvement, focusing largely on instrumental, nurturing, and school-related functions within Western cultural contexts. Similarly, the Chinese Parental Involvement and Support Scale for Preschool Children (CPISSPC) (Yue et al., 2022)was developed to evaluate parental involvement specifically in the early learning experiences of young children in China, centering primarily on support within academic or structured family settings. Despite their valuable contributions, these measures tend to confine parental involvement to observable and academic-oriented behaviors, particularly emphasizing parental guidance with schoolwork, rules, or educational expectations. This approach often neglects broader psychological and relational aspects of involvement, such as emotional support, everyday parent–child interactions, and social or psychosocial guidance, all of which are known to play a fundamental role in shaping adolescent development (Benner et al., 2016; Pinquart and Ebeling, 2020). Furthermore, these instruments frequently rely on retrospective reports from adult children or parental self-reports, and their constructs are often based on Western theoretical models, limiting both their cultural sensitivity and cross-cultural applicability. As a result, these tools may not adequately capture the subjective, lived experiences of adolescents, particularly in non-Western contexts such as China, where parenting practices are deeply influenced by unique cultural values, family dynamics, and social expectations.

Recent investigations have primarily examined the concept and effects of parental involvement by the perspectives from educational sciences, yet there remains a paucity of research from a psychological perspective that delves into the underlying psychological connotations and characteristic dimensions of this social phenomenon. Previous studies typically exhibit certain limitations: (1) a narrow focus on parental engagement in children’s learning activities within familial or school settings (Benner et al., 2016); and (2) inadequacies in measurement tools, which tend to emphasize educational involvement while neglecting the emotional, life, and social aspects of parental engagement (Benner et al., 2016; Pinquart and Ebeling, 2020). As a result, these tools often fail to accurately capture the complexity of parental involvement and its specific influence on adolescent development (Benner et al., 2016). To address these gaps, the present study seeks to explore the multidimensional characteristics of parental involvement as perceived by adolescents, utilizing a psychological perspective. As aforementioned, we hypothesize that parental involvement could be constituted by four main ingredients, at least, including academic, life, social and emotional involvement. By integrating qualitative research with psychometric methodologies, the research aims to construct a comprehensive framework that encapsulates the psychological underpinnings and characteristic dimensions of adolescents’ perceptions of parental involvement within the unique cultural context of Chinese families. Based on this integrative theoretical framework, a new hypothesis emerges: a structural scale to measure these multidimensional conceptions underlying the parental involvement. Furthermore, this framework will undergo validation across diverse samples to furnish a solid theoretical foundation and effective instruments for future research. The study is structured as follows:(1) identifying the dimensions underlying of adolescents’ perception of parental involvement in China;(2) Generating an initial pool of items and developing an initial scale, followed by exploratory and confirmatory factor analysis to verify the stability of the psychological structure about adolescents’ perception of parental involvement; (Tumasian et al., 2023) reliability and validity testing of the scale. The studies involving humans were approved by the IRB of the Nanchong Psychosomatic Hospital. The studies were conducted in accordance with the local legislation and institutional requirements. Written informed consent for participation in this study was provided by the participants’ legal caregivers. Given no designs to clinical trials in the present study, no registration is rendered.

## Study 1 constructing dimensions of adolescents’ perceptions of parental involvement through grounded theory

The purpose of this study is to explore adolescents’ perceptions of parental involvement using qualitative research methodologies. By employing grounded theory as the analytical framework, we conducted in-depth interviews with middle school students in China to collect primary data on their views and interpretations of parental involvement. Subsequently, the collected textual data have undergone systematic coding and analysis to explore the characteristic dimensions of adolescents’ perceptions of parental involvement.

### Methods

#### Development of the interview guidance

This study principally employs interviews as the primary method for data collection, supplemented by literature review and online materials. To ensure participants possess a clear and accurate understanding of the interview content, the research team—which includes two psychology professors, two doctoral candidates, and one middle school psychology teacher—engaged in discussions to draft a preliminary interview guide. Three participants were selected for pilot interviews to test the guide, after which modifications were made based on the outcomes and participant feedback. This process resulted in the final interview guide, which includes an introduction to the research background and core interview questions such as: “In what ways do you perceive your parents as involved in your development? Please provide specific examples.” “What specific behaviors characterize their involvement in these areas?” and “How has their involvement impacted you?” These questions are designed to elicit comprehensive insights into adolescents’ perceptions of parental involvement.

#### Participants

This study employs theoretical sampling to select participants, focusing on in-school adolescents from Chongqing and Shanxi in China, resulting in a total sample size of 61 individuals. The participant group consists of 20 middle school students (10 females, Mage = 13.90 ± 0.79) and 41 high school students (22 females, Mage = 16.93 ± 0.79). Among these participants, 72.13% reside in school dormitories, and 85.25% come from two-parent households. This sampling strategy ensures a diverse representation of adolescents for a comprehensive exploration of their perceptions of parental involvement.

#### Structural interview process and data collection

Before conducting the in-depth interviews, an interview team was established. This team comprised two master’s students and three doctoral candidates in psychology, as well as one doctoral candidate in education. Six interviewers were trained, guided, and coordinated both at the outset and throughout the interview process. Approximately 1 week before the interviews, the team communicated with school counselors to provide an overview of the interview content, enabling them to inform the students and agree on the interview method, timing, and location to ensure participant comfort. The formal interviews were conducted on a one on one basis to minimize participant apprehension, with audio recordings made following participant consent. The interviews, averaging approximately 20 min in duration with the longest extending to 36 min. After each interview, the research team promptly transcribed the recordings. The interview process was temporarily suspended upon reaching a point where no novel concepts appeared to be emerging, indicating potential data saturation. Detailed transcripts were then prepared and coded. Based on the coding results, the team decided whether to resume interviews, continuing this iterative process until saturation was achieved and no new concepts emerged. In total, approximately 190,000 words of interview transcripts were compiled.

#### Grounded theory

Grounded theory is regarded as a relatively scientific methodology within qualitative research, effectively addressing issues such as the lack of methodological rigor, difficulty in tracing and verifying research processes, and weak persuasive power of conclusions. As one of the three major schools of grounded theory, classic grounded theory emphasizes avoiding any preconceived assumptions during the research process, allowing theories inherent in reality to naturally emerge. It is regarded as having the most empirical character (Xudong, 2016). The purpose of this study is to deeply analyze the structural dimensions and impact of adolescents’ perceptions of parental involvement. Given the mixed evaluations in existing research on adolescents’ perceptions of parental involvement, this study adopts the more objective and scientific classic grounded theory as its primary research method.

#### Data analysis

This study utilized NVivo 14.0 (N14), a qualitative analysis software, to organize, edit, code, and perform statistical analysis on the collected interview data (Dhakal, 2022). Although N14 does not automate the entirety of the qualitative analysis or complete all coding processes independently, it is instrumental in facilitating the indexing, searching, and theorizing of unstructured and non-numeric textual data by importing Chinese interview transcripts. This process aids researchers in establishing indexes, identifying logical relationships, and generating theories from the data. Leveraging the N14, we conducted an open coding, which involves the process of breaking down textual data into distinct nodes based on a comprehensive understanding of the discourse within the written materials (Chen, 1999). This method disassembles the text into meaningful units through coding, transforming the data into different nodes. Using N14, the imported textual data—including words, sentences, and paragraphs—are meticulously read and analyzed. The process focuses on “adolescents’ perceptions of parental involvement” as the central theme, continuously searching for and comparing recurring meaningful units, which are then designated as different nodes and coded accordingly. These nodes form the fundamental analytical units within the data analysis process. The axial and selective coding processes were further applied to the textual data concerning “adolescents’ perceptions of parental involvement.” This involved reclassifying, refining, and synthesizing the codes that had already been obtained, merging codes with similar meanings, and organizing the interrelationships among the codes. Axial coding refers to the process by which researchers use the most significant or frequently occurring open codes to deeply categorize, synthesize, and organize textual data, thereby identifying codes with semantic relationships and linking them to each other (Chen, 1999).

### Results

#### Open coding

As shown in Table 1, using open coding approach, this study identified a total of 37 codes, encompassing 655 reference points.

**Table 1:** Distribution of coding nodes for adolescents’ perceptions of parental involvement.

Codes	Open coding	Reference points (n)	Axial coding (category)	Selective coding (Tertiary coding)
1	Expectation for achieving specific ranks	21	Academic expectations	Academic involvement
2	Requirement to attain certain scores	17		
3	Monitoring fluctuations in academic performance	13		
4	Providing encouragement after good academic performance	12		
5	Offering criticism or encouragement following poor performance	9		
6	Supervising study	45	Academic supervision	
7	Checking homework	16		
8	Providing tutoring or supplementary lessons	30		
9	Applying pressure to excel academically	9		
10	Inquiring about academic progress	7	Educational communication	
11	Obtaining insights from teachers	3		
12	Communicating the importance of education	16		
13	Establishing dress code requirements	7	Health management	Life involvement
14	Ensuring physical health	14		
15	Promoting dietary health	42		
16	Maintaining personal safety	4		
17	Regulating smartphone usage	20	Life rules	
18	Managing daily schedules	19		
19	Enforcing family rules	15		
20	Adjusting moods	4	Life interaction	
21	Assigning household chores	13		
22	Engaging in interactive activities	44		
23	Advising against friendships with certain types of individuals	18	Friendship supervision	Social involvement
24	Recommending friendships with specific individuals	2		
25	Opposing relationships with the opposite sex	10		
26	Supervising and scrutinizing friendship activities	23		
27	Teaching children how to resolve conflicts with peers	9	Friendship guidance	
28	Acceptance and support of children’s friends	6		
29	Educating and modeling appropriate social interactions	20		
30	Providing support and assistance	15	Emotional support	Emotional involvement
31	Offering encouragement	27		
32	Demonstrating respect	32		
33	Delivering positive feedback	16		
34	Showing respect and understanding	26	Emotional communication	
35	Sharing emotions and daily life experiences	15		
36	Desiring more emotional communication	38		
37	Practicing active listening	18		
	Total	655		

#### Axial and selective coding

Given that the 37 codes derived from open coding represent a listing of different meaningful units, they require systematic organization and analysis. By employing methods such as similarity comparisons, difference comparisons, lateral comparisons, and longitudinal comparisons, these 37 codes were consolidated into 10 more refined codes. Subsequently, selective coding involves a more abstract integration and refinement of existing categories to generate and explore core categories related to the primary theme. Centered on “adolescents’ perceptions of parental involvement,” four new nodes were established based on the 10 refined codes: “academic involvement,” “life involvement,” “social involvement,” and “emotional involvement” (Table 1). This process helps to construct and explore the links between categories, providing a deeper understanding of the theme. Specifically, emotional support emerges as the core category of parental involvement. Respondents expressed a desire for greater emotional support from their parents, including empathy and emotional communication. The interviewed adolescents generally perceived that their parents were involved in various aspects of their growth, including academics, daily life, social interactions, and emotions. Examples of interview data across these dimensions are presented in Table 2.

**Table 2:** Interview examples for each dimension of adolescents’ perceptions of parental involvement.

Dimension	Source Semantic Example
Academic involvement	“When I get home, they supervise or check if I’ve done my homework. After I finish, they just look it over to see if it’s all completed.”
	“After coming home from school, if they see me doing nothing, they want me to study. On weekends, they check my homework and stuff.”
	“My mom enrolls me in tutoring classes and also teaches me how to read.”
Life involvement	“I have to stick to eating breakfast every day, and after dinner, we usually have some fruit.”
	“They remind me about fire safety, treating me like I’m three, and they keep talking about fire safety precautions.”
	“If I wake up early, they’ll just say a few more words. But I usually sleep late, and in the morning, because of school, I have a fixed schedule. Early to bed, early to rise, no staying up late.”
Social involvement	“They tell me not to be friends with people who do not study seriously or have poor character.”
	“Sometimes when I go out to play, they ask who I’m with and how long I’ll be out.”
	“When I have conflicts with classmates, I sometimes hold grudges, but my father tells me to reconcile and guide me on how to make up with friends.”
Emotional involvement	“Whether I do well or badly in exams, they do not scold or hit me, they just encourage me.”
	“My father’s education level is pretty low, and when I try to explain things I learned to him and he does not get it, he starts to get impatient and scolds me, then I get impatient and shout back. He then tries to suppress me morally.”
	“I talk about things that happened at school, and he shares some of his day’s experiences with me.”
	“I feel there’s still a generational gap between us. I actually want more of a friendship-like exchange with him.”

#### Theoretical model construction

**Figure 1:**
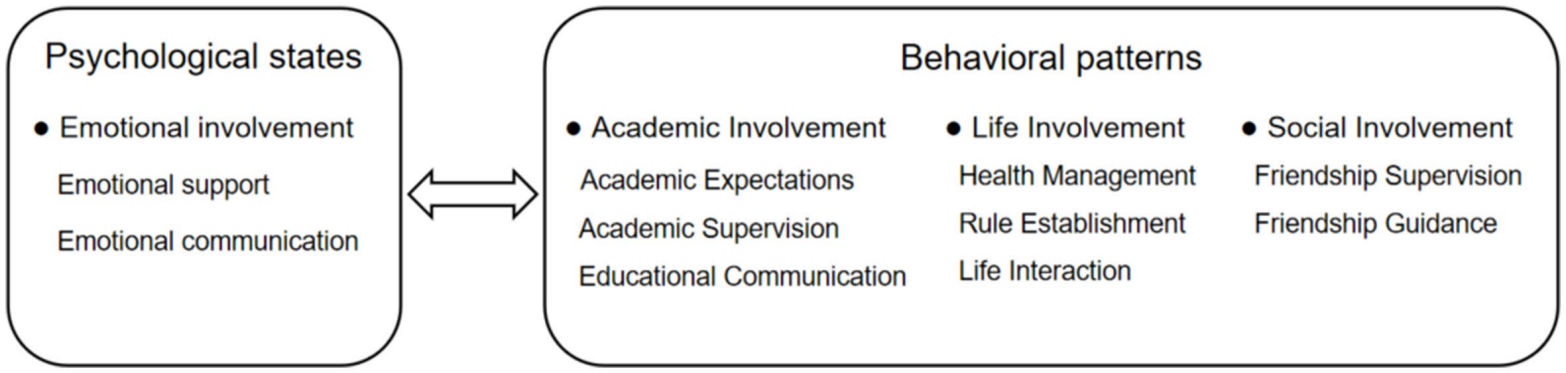
Theoretical construction of adolescents’ perception of parental involvement.

Based on the existing interview data and coding results, the researchers employed a bottom-up approach to gradually construct the psychological connotations of adolescents’ perceptions of parental involvement (Shueman and Wolman, 2012). Family systems theory provides a framework for understanding the multidimensional perceptions of parental involvement by adolescents, emphasizing the impact of family interaction patterns on psychological states and behavior patterns (Pascuzzo et al., 2013). Integrating our coding and comparison of emerging concepts, we identify four key dimensions of adolescents’ perceptions of parental involvement: emotional involvement, academic involvement, life involvement, and social involvement (Figure 1). In terms of psychological states, emotional involvement encompasses the emotional exchange and support between parents and adolescents, directly influencing adolescents’ emotional health and regulatory abilities. Regarding behavior patterns, academic, life, and social involvement reflect the guidance and expectation roles of parents within the family. Academic involvement influences adolescents’ academic performance, life involvement affects their lifestyle habits, and social involvement pertains to their social skills and networks. Utilizing this comprehensive perspective allows for a thorough examination of how parental involvement plays a pivotal role in the overall development of adolescents.

#### Theoretical saturation testing

Following the completion of the coding process, the research team conducted additional interviews with two participants. After organizing, coding, and analyzing these additional sources, no new conceptual categories emerged. Therefore, it was determined that the previously constructed dimensions of parental involvement have reached theoretical saturation.

## Study 2: development of the adolescents’ perceptions of parental involvement scale (PPIS)

This study aims to quantitatively validate the theoretical dimensions of adolescents’ perceptions of parental involvement that were initially identified using interviews in conjunction with grounded theory. The qualitative research led to the extraction of four characteristic dimensions of adolescents’ perceptions of parental involvement. Building on this foundation, the current study seeks to assess the stability and reliability of these qualitative findings through quantitative research. Therefore, based on the characteristic dimensions identified in Study 1 using grounded theory, we aim to develop a measurement questionnaire for adolescents’ perceptions of parental involvement using an open survey approach. This further refined and explored the characteristic dimensions of adolescents’ perceptions of parental involvement, ensuring the questionnaire’s validity and reliability.

### Method

#### Items determination

The initial development of the questionnaire for measuring adolescents’ perceptions of parental involvement was informed by the conceptual framework derived from grounded theory and detailed interview coding data. Drawing on established scale items, preliminary items for the questionnaire were crafted to reflect the identified dimensions of parental involvement. Subsequently, five psychology professors, eight associate professors in psychology, and 20 secondary school psychology teachers were invited to review the drafted questionnaire items and their phrasing. This review ensured alignment between the items and the theoretical dimensions. Incorporating expert feedback, the questionnaire was refined to contain a total of 84 items across five dimensions, including 24 items for academic involvement, 22 items for life involvement, 17 items for social involvement, and 21 items for emotional involvement. To enhance the clarity and accuracy of the questionnaire, an additional evaluation was conducted with 30 eighth-grade students, who assessed its readability. Based on their feedback, further revisions were made to the wording of the items.

#### Participants

In this study, we employed a convenient cluster-sampling method to ensure diverse data collection from various regions across China as far as possible. Specifically, data were gathered from 12 schools located in the following provinces: Heilongjiang in the Northeast, Shanxi in the North, Zhejiang and Jiangxi in the East, Guangdong in the South-Central area, Sichuan in the Southwest, and Shaanxi in the Northwest. The involvement of schools from diverse geographical areas was intended to enhance the representativeness and generalizability of our findings regarding adolescents’ perceptions of parental involvement. To collect data, paper-based questionnaires were distributed by trained school psychologists to maintain consistency and reliability in data collection procedures. A total of 2,290 responses were initially collected. Inclusion are specialized herein: (1) healthy adolescents who are undergoing school educations; (2) adherence to fill complete questionnaires. However, after excluding responses that demonstrated patterns of inattentive or random answering, the final dataset comprised 2,062 valid responses, resulting in an effective response rate of 90.04%. Exclusion criteria for “inattention” and “randomness” are as follow: (1) missing data over 20% of items; (2) selecting identical responses across all items (e.g., endorsing “Strongly Agree” for every Likert-scale question); (3) alternating between options in a fixed sequence (e.g., A-B-C-A-B-C) or extreme values (e.g., all “1″ or “5″ on a 5-point scale); (4) exclusively selecting neutral/middle options (e.g., “Neither Agree nor Disagree”). The sample consisted of 1,127 female participants, accounting for 54.66% of the total sample. The average age of participants was 14.99 years, with a standard deviation of 1.81 years, reflecting a typical adolescent age range. The grade distribution was as follows: 278 students were in the first year of middle school (13.48%), 410 in the second year (19.88%), 297 in the third year (14.41%), 462 in the first year of high school (22.41%), 197 in the second year (9.55%), and 418 in the third year of high school (20.27%). Additionally, the data revealed that 86.2% of the respondents came from two-parent families, a variable that may influence adolescents’ perceptions of parental involvement.

#### Statistical analysis

Two thousand and sixty-two participants completed the initially developed adolescents’ perceptions of parental involvement questionnaire, totaling 84 items. SPSS 24.0 software was used to item analysis and exploratory factor analysis.

### Results

#### Item analysis

The total scores of the scale were ranked, with the top 27% of the sample constituting the high-score group and the bottom 27% forming the low-score group. An independent samples t-test was conducted to analyze score differences across all items between these two groups, revealing that Item 17 did not show a significant difference between groups. Additionally, a correlation analysis was performed between each item score and the total scale score. The results indicated that the correlation coefficients (r) for Items 4, 10, and 72 were 0.24, 0.23, and 0.35, respectively, all of which were below 0.40. Consequently, these items were removed, resulting in a final set of 80 items.

#### Exploratory factor analysis (EFA) and model modification

The Bartlett’s test of sphericity [*x*^2^ = 22970.807, df = 210, *p* < 0.001] and the Kaiser-Meyer-Olkin (KMO) test (KMO = 0.948) indicated that the items were suitable for exploratory factor analysis (EFA) due to the likelihood of shared underlying factors. Principal component analysis with an oblique rotation was employed to extract factors with eigenvalues greater than 1. Factors and items were screened based on the following criteria: ① factor loadings < 0.45; ② commonalities < 0.2; ③ extracted factors with fewer than 3 items; ④ items displaying cross-loadings; ⑤ items that were ambiguous or clearly misclassified (Watkins, 2018). EFA was repeated each time an item was removed until no items met any of the aforementioned conditions. After eliminating 59 items, the final questionnaire retained 21 items distributed across four factors, with a cumulative variance contribution rate of 64.337%. The factor loadings for all items on their respective factors ranged from 0.561 to 0.943 (Table 3). As a result, the exploratory factor analysis yielded a measurement questionnaire for parental involvement perception comprising 21 items. These include 9 items for the emotional involvement dimension, 5 items for the social involvement dimension, 4 items for the health involvement dimension, and 3 items for the academic involvement dimension.

**Table 3:** The 4 characteristic dimensions of the adolescents’ perception of parental involvement questionnaire.

Test items	EI	SI	LI	AI	Communality
When I face difficulties, my parents encourage me and boost my confidence, making me more resilient.	0.873				0.699
My parents praise my efforts and achievements, regardless of the outcome, providing positive feedback and encouragement.	0.867				0.735
My parents regularly communicate with me to understand my feelings and emotions, offering their support.	0.864				0.637
My parents try to see things from my perspective to better understand my feelings.	0.814				0.697
My parents understand the emotional challenges I face with friends, at school, or within the family.	0.813				0.693
My parents listen attentively when talking to me.	0.786				0.66
When I feel frustrated or sad, my parents comfort me promptly and help me manage my emotions.	0.772				0.671
My parents guide me on how to properly face and handle negative emotions, such as anger or anxiety.	0.754				0.458
My parents respond to my emotional expressions with empathy and comfort.	0.74				0.687
My parents teach me how to choose trustworthy and supportive friends.		0.822			0.571
My parents invite my friends over to our house.		0.771			0.604
My parents inquire about and keep track of the specific times and places of my outings with friends.		0.762			0.578
My parents ensure that I follow family safety rules when going out, such as coming home on time and staying in touch.		0.731			0.567
My parents evaluate my friends and share their opinions and suggestions with me.		0.667			0.554
My parents supervise and encourage me to engage in regular physical exercise daily.			0.943		0.685
My parents take me for regular medical check-ups.			0.776		0.681
My parents ensure that our daily diet is healthy and balanced.			0.674		0.575
My parents limit my consumption of junk food.			0.561		0.539
My parents provide me with additional learning resources, such as tutoring classes or online courses.				0.902	0.748
My parents supervise my participation in extracurricular tutoring or remedial classes.				0.88	0.783
My parents frequently urge and tutor me in my studies.				0.732	0.69
Rotated eigenvalues	8.605	2.37	1.36	1.176	
Variance explained	40.98%	11.28%	6.48%	5.60%	64.34%
Internal consistency reliability	0.929	0.803	0.784	0.819	0.922

## Study 3: validation of the adolescents’ perceptions of parental involvement scale (PPIS)

In Study 3, an investigation was conducted using the PPIS developed from Study 2 on an additional sample of 2,060 middle school students. Confirmatory factor analysis (CFA) was employed to ascertain the potential dimensions underlying adolescents’ perception of parental involvement. In this process, construct validity, criterion-related validity, and internal consistency reliability were assessed, alongside evaluating fit indices to ensure the robustness of the model (LU et al., 2009).

### Methods

#### Participants

Data for the study were collected through a convenient cluster-sampling method across 12 schools located in various regions of China, namely Heilongjiang in the northeast, Shanxi in the north, Zhejiang and Jiangxi in the east, Guangdong in the south-central area, Sichuan in the southwest, and Shaanxi in the northwest. A total of 2,200 paper-based questionnaires were distributed by psychology teachers, out of which 2,060 were deemed valid after removing patterned and careless responses, resulting in an effective response rate of 93.64%. The sample consisted of 1,102 female students (53.50%), with an average age of 14.86 years (SD = 1.87). The grade distribution was as follows: 301 seventh graders (14.61%), 469 eighth graders (22.77%), 290 ninth graders (14.07%), 418 tenth graders (20.29%), 223 eleventh graders (10.83%), and 359 twelfth graders (17.43%). Notably, 86.1% of the students were from two-parent families. For Criterion-related validity assessment, a separate cluster sampling was carried out in five schools located in Shanxi, Hebei, Sichuan, and Chongqing. Out of 4,000 distributed questionnaires, 3,813 were returned and valid, yielding a response rate of 95.33%. This sample comprised 2,042 female students (56.55%) with an average age of 15.18 years (SD = 1.81). The grade distribution was 20.15% for seventh graders, 4.51% for eighth graders, 3.49% for ninth graders, 29.56% for tenth graders, 31.68% for eleventh graders, and 10.25% for twelfth graders.

#### Measurements

Here, this PPIS developed in the Study 2 is used as the measurement for being validated, which encompassed of 21 initial items. To examine the criterion-related validity, we included the Parental Expectations Questionnaire, which initially developed by Lu (2014), consisting of 24 items covering five dimensions: academic expectations, future achievements, behavioral expectations, interpersonal relationships, and physical and mental qualities. The questionnaire employs a five-point Likert scale ranging from 1 (not at all applicable) to 5 (very applicable). The total score is derived from the sum of four dimensions, with higher scores indicating higher levels of parental expectations. In this study, the Cronbach’s alpha coefficient for the ASMPQ was 0.746, indicating acceptable internal consistency.

#### Confirmatory factor analysis (CFA)

AMOS 27.0 software was used to analyze the PPIS obtained from Study 2. Based on the common evaluation index requirements in psychometric questionnaire development, the selected indicators include χ2/df, root mean square error of approximation (RMSEA), standardized RMR (SRMR), Comparative Fit Index (CFI) and TuckerLewis Index (TLI).

### Result

#### Confirmatory factor analysis

**Figure 2:**
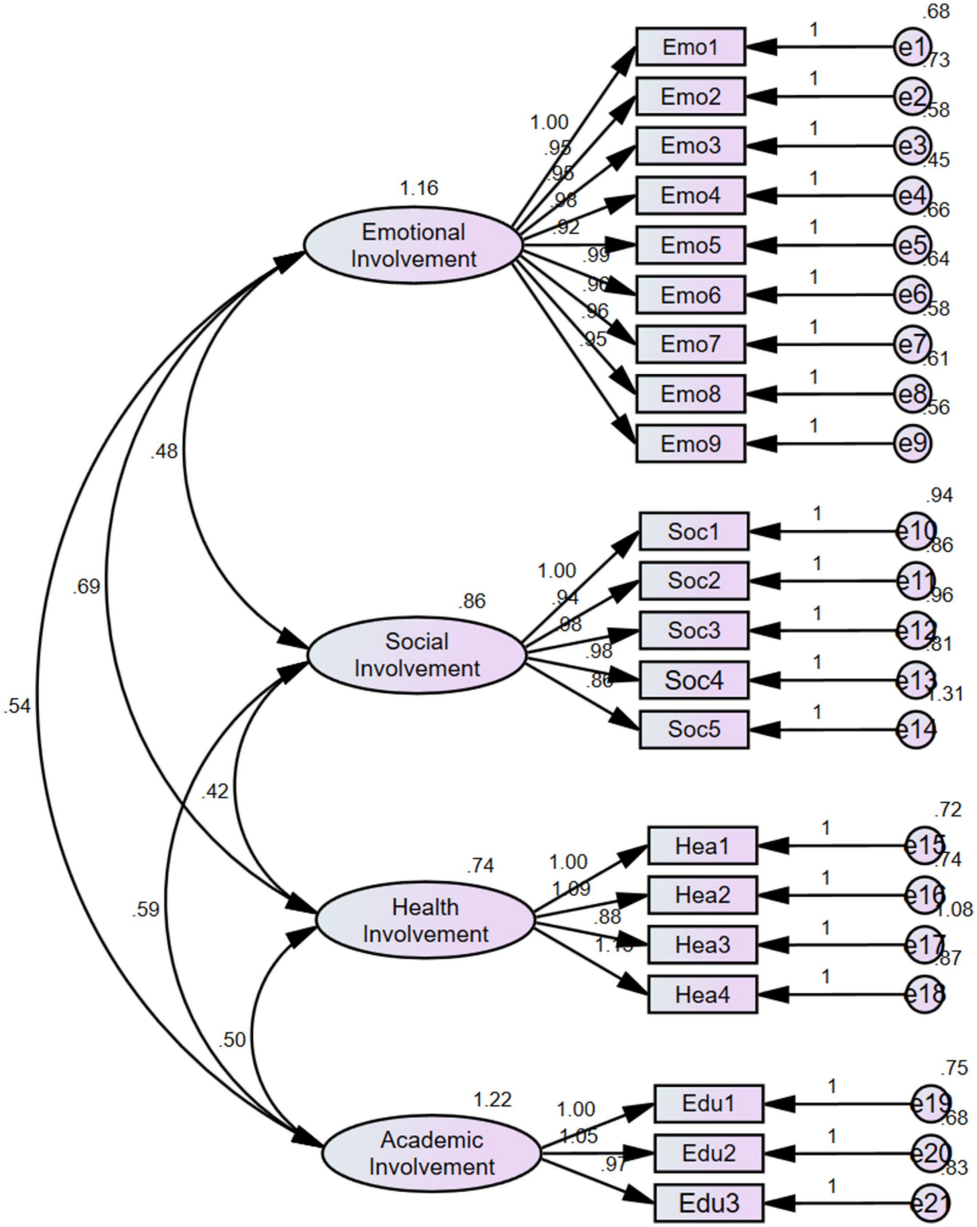
The results of CFA.

To further validate the four-dimensional structure derived from the exploratory factor analysis, a confirmatory factor analysis (CFA) was conducted using AMOS 27.0 (Collier, 2020). The results indicated that the fit indices for the four-dimensional model of adolescents’ perceptions of parental involvement were within acceptable ranges (Table 4 and Figure 2). Additionally, attempts were made to reduce the dimensions, but comparative analyses demonstrated that the fit indices of the four-dimensional model were significantly superior to those of other models.

**Table 4:** Comparison of confirmatory factor analysis results for adolescents’ perception of parental involvement.

	χ^2^	df	χ^2^/df	CFI	TLI	SRMR	RMSEA
Four-factor model	1265.867^***^	183	6.917	0.955	0.948	0.081	0.054
Three-factor model	2686.843^***^	186	14.445	0.895	0.882	0.116	0.081
Two-factor model	3784.234^***^	188	20.129	0.849	0.832	0.143	0.096
One-factor model	5955.594^***^	189	31.511	0.758	0.732	0.189	0.122

Four-Factor Model: Emotional Involvement, Social Involvement, Health Involvement, Academic Involvement; Three-Factor Model: Emotional Involvement, Social Involvement, Health Involvement + Academic Involvement; Two-Factor Model: Emotional Involvement, Social Involvement + Health Involvement + Academic Involvement; One-Factor Model: Emotional Involvement + Social Involvement + Health Involvement + Academic Involvement. ^**^p < 0.01, ^***^p < 0.001.

#### Construct validity

Construct validity was evaluated through convergent and discriminant validity. The results indicated that the correlation coefficients between each dimension and the total score ranged from 0.664 to 0.890 (*p* < 0.001), whereas the inter-correlations among the four dimensions ranged from 0.399 to 0.580 (*p* < 0.001). The higher correlations between the total score and each dimension, as compared to those between the subscales themselves, support strong convergent validity, suggesting that all dimensions measure the same construct. The moderate correlations among dimensions indicate that the construct can be assessed distinctly across different dimensions, demonstrating good discriminant validity. Furthermore, the composite reliability (CR) exceeded 0.80, standardized factor loadings ranged from 0.573 to 0.844 (all greater than 0.50), and the average variance extracted (AVE) ranged from 0.458 to 0.783 (all greater than 0.40), all of which reached acceptable levels (Collier, 2020). These findings further corroborate the strong convergent validity of the scale, confirming that it reliably measures the intended construct.

#### Criterion-related validity

Criterion-related validity was assessed by calculating the correlation between parental expectation scores and the scores of the Adolescents’ Perception of Parental Involvement Scale. The total score and each factor score of the Adolescents’ Perception of Parental Involvement Scale demonstrated positive correlations with the parental expectation scale (Table 5). This finding indicates that the questionnaire possesses strong criterion-related validity and that both scales measure similar traits.

**Table 5:** Correlation between adolescents’ perception of parental involvement and criterion measures (*n* = 3,813).

Criterion Measures	Parental Involvement	Emotional Involvement	Social Involvement	Health Involvement	Academic Involvement
Parental expectations	0.434^**^	0.376^**^	0.219^**^	0.402^**^	0.223^**^

^**^p < 0.01, ^***^p < 0.001.

#### Internal reliability

The Cronbach’s αfor the total score of PPIS, as well as for the dimensions of academic involvement, health involvement, social involvement, and emotional involvement, ranged from 0.785 to 0.940. The Guttman split-half reliability is 0.836, All of these values exceed the threshold of 0.700, indicating acceptable internal consistency reliability (Collier, 2020). This suggests that the scale is a reliable measure of the various facets of perceived parental involvement.

In summary, this PPIS developed above showed well psychometric performance, including internal reliability as well construct and criterion-related validity. Thus, the formal PPIS has been provided in the Supplementary material.

## Discussion

This research explored adolescents’ perceptions of parental involvement through two complementary research processes. Study 1 employed grounded theory along with qualitative interviews to construct a conceptual framework of parental involvement encompassing four dimensions: emotional, academic, life, and social. This framework reveals how these multidimensional modes of family involvement impact adolescents’ psychological states and behavior patterns. Building on these insights, Study 2 developed and validated the PPIS. Through rigorous item analysis, exploratory factor analysis (EFA), Study 3 ensure the scale’s validity and reliability.

### Concept structure of the adolescents’ perceptions of parental involvement

In this study, we employed a bottom-up approach grounded in the principles of grounded theory to construct a comprehensive psychological framework of adolescents’ perceptions of parental involvement. Family systems theory provided a foundation for understanding the multidimensional nature of parental involvement as perceived by adolescents, emphasizing the influence of familial interaction patterns on psychological and behavioral dimensions (Shueman and Wolman, 2012). We identified four core dimensions—emotional, academic, life, and social—in this multifaceted construct. Emotional involvement focuses on the exchange and support between parents and adolescents, playing a critical role in shaping adolescents’ psychological health. In terms of behavior patterns, academic, life, and social involvement reflect parental roles in guiding the family. Academic involvement enhances adolescents’ academic performance through supervision and support; Life involvement influences their habits through regular management and guidance; Social involvement aids in developing effective social skills and networks. These behavioral dimensions indicate that parental involvement significantly impacts adolescents across all areas of life.

Emotional involvement emerged as a crucial dimension in our study. According to attachment theory (Lowenstein, 2010), parental emotional support is key to establishing secure attachments, which fosters positive self-concept and coping strategies in adolescents. Research indicates that emotionally supported adolescents exhibit greater confidence and adaptability in the face of challenges, possessing enhanced emotional regulation abilities (Farsijani et al., 2022; Pascuzzo et al., 2013). Our open coding highlighted that adolescents rely on parental emotional support as a resource for alleviating stress, enabling them to manage emotions and solve problems more effectively. This emotional involvement not only provides short-term emotional stability but also lays the groundwork for long-term psychological health, directly influencing adolescents’ emotional regulation capabilities (Ren et al., 2024).

Research suggests that parental involvement in managing and guiding children’s learning positively impacts academic achievement (Xiong et al., 2021). Social learning theory posits that parents serve as significant figures in learning, modeling attitudes and behaviors that foster academic engagement (Sonnenschein et al., 2012). Our study found that adolescents perceive parental involvement in tasks such as homework assistance, resource provision, and creating conducive learning environments, which bolster enthusiasm and commitment to learning. The positive effects of academic involvement may stem from enhancing adolescents’ academic self-efficacy and intrinsic motivation. Existing literature notes that self-efficacy encourages students to set higher academic goals and maintain persistence (Honicke and Broadbent, 2016; Schunk and DiBenedetto, 2020). Thus, academic involvement serves not just as an academic focus but as a means to cultivate independent learners. Our findings indicate parental participation in daily activities such as diet and routines promotes healthy lifestyle habits. Prior research corroborates that regular family activities and clear behavioral expectations contribute to stable life patterns, positively affecting adolescents’ physiological and psychological well-being (Spagnola and Fiese, 2007; Walsh, 2016). Our results emphasize parents’ roles in shaping adolescents’ social skills and networks. Emotional socialization theory suggests that parental guidance in social settings aids adolescents in navigating the complexities and challenges of social relationships (Fosco and Lydon-Staley, 2019). Adolescents in our study reported that parental advice, social restrictions, and encouragement of positive friendships help guide their social engagements. Research indicates that such social guidance not only reduces antisocial behaviors but also enhances social competencies and emotional intelligence (Mounts, 2011).

This comprehensive perspective enhances our understanding of the critical role parental involvement plays in adolescent development, providing a solid theoretical foundation for future research and interventions. The insights from this theoretical construction deepen our understanding of family influences on adolescents and offer new possibilities for enhancing parent–child interaction quality and educational strategies.

### Reliability and validity of the PPIS

In previous research, many tools examining parental involvement have predominantly focused on academic and school-related activities, such as attending parent-teacher meetings, monitoring homework completion, and communicating with teachers (Grolnick and Slowiaczek, 1994; Lara and Saracostti, 2019; Park and Holloway, 2013; Pek and Mee, 2020). These assessments are often filled out by parents, which may not accurately capture adolescents’ subjective perceptions of parental involvement (Fantuzzo et al., 2000; Lara and Saracostti, 2019; Pek and Mee, 2020; Walker et al., 2005). Adolescence is a pivotal transition period from dependency to independence, during which adolescents’ understanding and perceptions of parental involvement can significantly differ from those of their parents (Liu and Zhang, 2023). Adolescents often place more emphasis on emotional and social support rather than just academic assistance (Branje, 2018). As their autonomy, reflexivity, and self-concept develop, adolescents interpret parental actions in more complex and diverse ways (Keijsers and Poulin, 2013). Therefore, obtaining feedback directly from adolescents allows for a more authentic and comprehensive understanding of parental involvement.

The PPIS developed in this study addresses this gap by expanding the concept of parental involvement to encompass emotional, life, and social dimensions, in addition to learning behaviors. This comprehensive approach examines the roles parents play not only in academic support but also in life guidance and emotional interaction. In validating the scale, we observed correlations between the emotional and social involvement dimensions and adolescents’ overall development, which aligns with existing literature emphasizing adolescents’ emotional and social needs (Tomás et al., 2020). The scale demonstrated strong validity and reliability in testing, with a particularly noteworthy positive correlation with the Parental Expectations Questionnaire, indicating its convergent validity. This tool provides a new instrument for both theoretical research and practical application, effectively capturing the nuanced differences in how adolescents perceive parental involvement. It is particularly valuable for developing personalized educational strategies, as the scale can identify specific areas that may require attention, enabling educators and counselors to offer more targeted support.

### Limitations and future research directions

Despite the comprehensive approach taken in this study, several limitations should be noted. First, the sample used in this study was drawn from a limited geographical area within China, which may limit the generalizability of the findings to other cultural or regional contexts. Future research should consider including a more diverse sample that encompasses different regions and cultural backgrounds to assess the cross-cultural applicability of the PPIS. Second, while the use of self-reported measures from adolescents offers valuable insights into their perceptions, it also introduces potential biases such as social desirability and subjective interpretation. Future studies might incorporate multi-informant approaches, collecting data from parents, teachers, and peers, to triangulate and validate the findings more comprehensively. Finally, this study employed a cross-sectional design, which captures perceptions at a single point in time. Longitudinal studies are needed to examine how perceptions of parental involvement may change over time and to identify the long-term impacts on adolescents’ developmental outcomes. In addition, we did not assess the test–retest reliability of the newly developed scale, as no follow-up data were collected to evaluate the temporal stability of participants’ responses. This limits our ability to determine the consistency of the scale across different time points. Future research should address both the inclusion of longitudinal designs and the assessment of test–retest reliability to further establish the robustness and applicability of this measurement tool.

## Conclusion

This study provides an in-depth examination of adolescents’ perceptions of parental involvement, highlighting its multifaceted nature through both qualitative and quantitative methodologies. By employing grounded theory and developing a robust measurement scale, we identified key dimensions of parental involvement—emotional, academic, life, and social. The development and validation of the PPIS fill a critical gap in the existing literature, offering a tool that more accurately captures the diverse experiences and insights of adolescents. This advancement not only enhances our theoretical understanding of parental involvement but also provides practical implications for educational and family interventions.

## Data Availability

The raw data supporting the conclusions of this article will be made available by the authors, without undue reservation.
