# Supplementary material for: Development and validation of the adolescents’ perceptions of parental involvement scale in China
Source: Front Psychol. 2025 May 14;16:1529173. doi: 10.3389/fpsyg.2025.1529173 (PMC12118379; doi:10.3389/fpsyg.2025.1529173)
Supplement: Supplementary file 1 [file Supplementary_file_1.docx]

Supplementary Material

**Adolescents’ Perceptions of Parental Involvement Scale**

In order to gain a deeper understanding of adolescents' perception of parental involvement during their growth process, we have specially designed this questionnaire titled 'Perception of Adolescent Parental Involvement'. There is no right or wrong in the questionnaire, please answer based on your true feelings.

| **Test Items** | **Never** | **Occasionally** | **Moderate** | **Often** | **Always present** |
| --- | --- | --- | --- | --- | --- |
| 1. My parents ensure that our daily diet is healthy and balanced. | 1 | 2 | 3 | 4 | 5 |
| 1. My parents evaluate my friends and share their opinions and suggestions with me. | 1 | 2 | 3 | 4 | 5 |
| 1. My parents understand the emotional challenges I face with friends, at school, or within the family. | 1 | 2 | 3 | 4 | 5 |
| 1. My parents regularly communicate with me to understand my feelings and emotions, offering their support. | 1 | 2 | 3 | 4 | 5 |
| 1. My parents supervise my participation in extracurricular tutoring or remedial classes. | 1 | 2 | 3 | 4 | 5 |
| 1. My parents limit my consumption of junk food. | 1 | 2 | 3 | 4 | 5 |
| 1. My parents inquire about and keep track of the specific times and places of my outings with friends. | 1 | 2 | 3 | 4 | 5 |
| 1. My parents respond to my emotional expressions with empathy and comfort. | 1 | 2 | 3 | 4 | 5 |
| 1. My parents listen attentively when talking to me. | 1 | 2 | 3 | 4 | 5 |
| 1. My parents try to see things from my perspective to better understand my feelings. | 1 | 2 | 3 | 4 | 5 |
| 1. My parents supervise and encourage me to engage in regular physical exercise daily. | 1 | 2 | 3 | 4 | 5 |
| 1. My parents ensure that I follow family safety rules when going out, such as coming home on time and staying in touch. | 1 | 2 | 3 | 4 | 5 |
| 1. My parents praise my efforts and achievements, regardless of the outcome, providing positive feedback and encouragement. | 1 | 2 | 3 | 4 | 5 |
| 1. When I feel frustrated or sad, my parents comfort me promptly and help me manage my emotions. | 1 | 2 | 3 | 4 | 5 |
| 1. My parents invite my friends over to our house. | 1 | 2 | 3 | 4 | 5 |
| 1. My parents guide me on how to properly face and handle negative emotions, such as anger or anxiety. | 1 | 2 | 3 | 4 | 5 |
| 1. My parents take me for regular medical check-ups. | 1 | 2 | 3 | 4 | 5 |
| 1. My parents provide me with additional learning resources, such as tutoring classes or online courses. | 1 | 2 | 3 | 4 | 5 |
| 1. When I face difficulties, my parents encourage me and boost my confidence, making me more resilient. | 1 | 2 | 3 | 4 | 5 |
| 1. My parents teach me how to choose trustworthy and supportive friends. | 1 | 2 | 3 | 4 | 5 |
| 1. My parents frequently urge and tutor me in my studies. | 1 | 2 | 3 | 4 | 5 |

**Interview Outline on Adolescents' Perceptions of Parental Involvement**

Basic information：

1. Gender： 🞎 Boy 🞎 Girl
2. Age：___________________
3. Grade：___________________
4. Do you live on campus：🞎 Yes 🞎 No
5. Family structure： 🞎Two-parent family 🞎Single-parent family (mother)  🞎Single-parent family (father)  🞎Blended/reconstituted family 🞎Other

Dear Student,

Hello. We are conducting a research study on parental involvement, and the information you provide will be of great value to our research. We kindly ask you to answer the questions thoughtfully and honestly. Please be assured that the results of this interview will be used solely for academic research purposes. All of your personal information will be kept strictly confidential. Thank you very much for your support and cooperation.

1. In your opinion, in which areas do your father and mother participate most in your upbringing? (You may discuss your father and mother separately.)
2. Regarding the aspects you just mentioned, what specific actions or behaviors demonstrate your father’s/mother’s involvement? Could you provide some concrete examples?
3. Has this involvement influenced your attitudes or perspectives? If so, how?
4. Overall, what impact do you think this involvement has had on you? Do you feel that it has been mostly positive, neutral, or somewhat negative? Could you elaborate on your reasons or provide examples?
5. Throughout your growth, how do your father and mother typically engage in your development? Are there one or two particularly memorable instances you could share?
6. When you encounter difficulties or problems, what approaches do your father or mother usually take to help you address them?
7. What effect does this behavioral support or intervention have on you? (For example, do you find it positive, no effect, or negative? Why?)
8. When you experience emotions related to failure, setbacks, or achievements, how do your father and mother typically respond? How do you feel about their responses?
9. How do you think their responses affect you? Do you find their impact is mostly positive, neutral, or somewhat negative? Could you briefly explain your reasons or give an example?
10. Overall, in which areas would you prefer your father or mother to be more or less involved? If you could change the ways your parents participate, what are your expectations or suggestions?
11. Is there anything else about your parents’ involvement in your development—something particularly impressive or important that we have not asked about—that you would like to share?
